# Bipartite and tripartite *Cucumber mosaic virus*-based vectors for producing the *Acidothermus cellulolyticus* endo-1,4-β-glucanase and other proteins in non-transgenic plants

**DOI:** 10.1186/1472-6750-12-66

**Published:** 2012-09-21

**Authors:** Min Sook Hwang, Benjamin E Lindenmuth, Karen A McDonald, Bryce W Falk

**Affiliations:** 1 Department of Plant Pathology, University of California, One Shields Avenue, Davis, CA, 95616, USA; 2 Department of Chemical Engineering and Materials Science, University of California, One Shields Avenue, Davis, CA, 95616, USA; 3 Present address: Bayer HealthCare Pharmaceuticals, 800 Dwight Way, Berkeley, CA, 94710, USA

**Keywords:** *Cucumber mosaic virus*, Protein production, Endoglucanase, *Agrobacterium tumefaciences*, Viral vector, Transient protein expression, *Nicotiana benthamiana*

## Abstract

**Background:**

Using plant viruses to produce desirable proteins in plants allows for using non-transgenic plant hosts and if necessary, the ability to make rapid changes in the virus construct for increased or modified protein product yields. The objective of this work was the development of advanced CMV-based protein production systems to produce *Acidothermus cellulolyticus* endo-1, 4-β-glucanase (E1) in non-transgenic plants.

**Results:**

We used two new *Cucumber mosaic virus* (CMV)-based vector systems for producing the green fluorescent protein (GFP) and more importantly, the *Acidothermus cellulolyticus* endo-1, 4-β-glucanase (E1) in non-transgenic *Nicotiana benthamiana* plants. These are the inducible *CMVin* (CMV-based inducible) and the autonomously replicating *CMVar* (CMV-based advanced replicating) systems. We modified a binary plasmid containing the complete CMV RNA 3 cDNA to facilitate insertion of desired sequences, and to give modifications of the subgenomic mRNA 4 leader sequence yielding several variants. Quantitative RT-PCR and immunoblot analysis showed good levels of CMV RNA and coat protein accumulation for some variants of both *CMVin* and *CMVar*. When genes for E1 or GFP were inserted in place of the CMV coat protein, both were produced in plants as shown by fluorescence (GFP) and immunoblot analysis. Enzymatic activity assays showed that active E1 was produced in plants with yields up to ~ 11 μg/g fresh weight (FW) for specific variant constructs. We also compared *in vitro* CMV genomic RNA reassortants, and CMV RNA 3 mutants which lacked the C’ terminal 33 amino acids of the 3A movement protein in attempts to further increase E1 yield. Taken together specific variant constructs yielded up to ~21 μg/g FW of E1 in non-transgenic plants.

**Conclusions:**

Intact, active E1 was rapidly produced in non-transgenic plants by using agroinfiltration with the CMV-based systems. This reduces the time and cost compared to that required to generate transgenic plants and still gives the comparable yields of active E1. Our modifications described here, including manipulating cloning sites for foreign gene introduction, enhance the ease of use. Also, *N. benthamiana,* which is particularly suitable for agroinfiltration, is a very good plant for transient protein production.

## Background

Using plant viruses as vehicles for foreign protein production in plants offers many advantages over more traditional prokaryotic-based, and even over transgenic plant-based protein production systems. For example, plants are relatively easy and inexpensive to grow, plants are able to perform post-translational protein modifications (e.g. glycosylation) not possible with prokaryotes, and plant cells can secrete appropriately engineered proteins allowing for simplified product purification [1,2]. Transgenic plants engineered to produce desirable proteins offer some of these advantages, but engineered plants require substantial time, effort and cost to develop and do not offer flexibility for rapid change if modifications to the protein product are desired. By contrast, using plant viruses to produce desired proteins in plants allows for using non-transgenic plant hosts and if necessary, the ability to make rapid changes in the virus construct for increased or modified protein product yields.

*Cucumber mosaic virus* (CMV) is the one of the viruses that has been used for protein production in plants [3,4]. CMV has an extremely wide plant host range [5] which opens the door for using plants other than only *Nicotiana* spp. for producing proteins, and thus an optimized CMV-based protein production system would be very desirable. But CMV also has some potential drawbacks for foreign protein production. CMV has a tripartite single-stranded RNA genome and each genomic RNA is packaged separately within icosahedral capsids [5]. CMV genomic RNAs 1 and 2 encode the 1a and 2a proteins, respectively, which are involved in viral RNA replication [5,6]. RNA 2 also encodes a small protein called 2b, which affects virulence and is known to suppress the initiation of the plant defense, RNA silencing, and to play a role in promoting cell-to-cell movement [7]. RNA 3 also is bicistronic, encoding the cell-to-cell movement protein (MP) and the virion capsid protein (CP). All three CMV genomic RNAs are essential for the systemic plant infection and all five CMV-encoded proteins directly or indirectly affect the movement of CMV within the plant host [5]. Still, CMV genome segments 2 and 3 have been modified in some cases for insertion of specific sequences which can give foreign protein production in plants [4,8,9].

In our previous work we engineered a binary plasmid to contain modified complementary DNAs (cDNAs) representing the complete CMV tripartite genome, in which the CMV coat protein gene was replaced by the gene encoding α-1-antitrypsin [AAT] [9]. We deleted a region of the CMV RNA 1 leader sequence to ensure that the viral replicase was not able to replicate the truncated RNA 1 and since coat protein was lacking, infectious CMV was not generated thereby eliminating possible unwanted spread of the recombinant CMV. Furthermore, because one of the key CMV-encoded protein components of the viral replicase (1a) is under the control of a relatively tightly regulated chemically inducible promoter (the XVE inducible promoter [10]), recombinant viral amplicons were produced intracellularly only after addition of the inducer (β-estradiol). The high efficiency and specificity are among the major advantages of the XVE system, and thus it provides a potent tool for research in plant biotechnology.

Despite the advantage of having all CMV components on a single plasmid (e.g. ensuring that all CMV components are simultaneously introduced into the same cell) [9], the *CMViva* plasmid proved to not be easy for subsequent manipulation. Its size alone (28 kbp) made subsequent cloning manipulations difficult. Therefore, here we explored development of new CMV-based smaller-sized variants by separating genome components onto different plasmids to give a bipartite inducible (*CMVin*, CMV-based inducible system) and tripartite, autonomously replicating forms of CMV (*CMVar*, CMV-based advanced replicating system). We also assessed the effects of mRNA4 leader sequence variants and compared two CMV genotypes for their abilities to give *in planta* production of two proteins, the green fluorescent protein (GFP) and the *Acidothermus cellulolyticus* endo-1, 4-β-glucanase (E1), a cellulose degrading enzyme. This heat-stable, 56,000 MW well-studied endoglucanase has been produced previously in different species of transgenic plants [11-13], and is believed to have potential application for cellulose biomass conversion to sugars and use in biofuel production. Here we show that active E1 can be rapidly produced in non-transgenic plants by using agroinfiltration with the CMV-based systems. This reduces the time and energy required to generate transgenic plants and still gives the comparable yields of active E1 to those obtained previously by others.

## Methods

### Plants and photography

Three-week-old *Nicotiana benthamiana* plants and nine day old zucchini squash (*Cucurbita pepo* L. cv. Green Bush) plants were used for virus inoculations or agroinfiltration. Plants were photographed with a Cannon G6 digital camera equipped with a Tiffen Deep Yellow 15 filter. For photographing GFP expression, plants were illuminated with a hand-held long-wave UV lamp.

### Cloning and plasmid construction

In order to develop *CMVin* (CMV-based inducible system) and *CMVar* (CMV-based advanced replicating system), the gene-of-interest was inserted into the coat protein coding region of CMV-Q RNA 3 (GenBank:M21464) [14] to give p*CMVar* RNA 3. We modified the CMV RNA 3 intergenic region, which also gives rise to the mRNA 4 leader sequence, by PCR primer tagging to introduce additional restriction enzyme sites for easier cloning. This was done by PCR amplifying the CP coding region using tagged forward primers (EATG for sequence 2, HATG for sequence 6, PHATG for sequence 8, and CPfwd for the wild type leader sequence, Table 1) and the reverse primer (CPrev listed in Table 1), and GoFlexi Taq DNA polymerase (Promega Corp., Madison, WI, U.S.A.). Amplified fragments were transferred to pGEM-T Easy (Promega Corp., Madison, WI, U.S.A.) and sequences were verified. Plasmids were then digested by *Pst* I and *Tth*111 I, and the desired fragment was transferred to pQA3 [9] (Additional file 1: Figure S1). Then sequences containing the CaMV 35S (35S) promoter, RNA 3 and 35S terminator were PCR amplified using primers 35SPfwd and 35STrev (Table 1) and *Pfu* DNA polymerase (Stratagene, Agilent TechnologiesCompany, U.S.A), and the resulting fragments were ligated into the *Sma* I site of the mini binary vector, pCB301 [15] (Additional file 1: Figure S1). These were then used as the RNA 3 source for *CMVar* and *CMVin* variants. The higher producing constructs (containing the 2, 6, and 8 modified, and wildtype leader sequences of RNA4; Additional file 2: Figure S2) were selected for further experiments.

**Table 1:** List of primers used here

**Primer ID**	**Sequences 5′-3′**
realRna3onlyfwd	AACAGATTAGCCGAGCATTCG
realRrna3rev	AGCTAACGTTGTTTAACTGCGACTT
realRna4onlyfwd	AGTGCCTTCATCATCCGATCTT
realRna4onlyrev	GGTGAGTTACCATCGCCAAAC
real18Sfwd	TACGCCCCGCCCAAA
real18Srev	CACTGGCAGTCCTTCGTGAGT
RNA3end	TGGTCTCCTTATGGAGAACCTGTGG
realRna1fwd	TGGTCAGTATGCCCCAAAGG
realRna1rev	TTCAAGGGTAGCTCGACAACCT
realRna2fwd	CCCAGACTGATGTCTCCCAAA
realRna2rev	GAGTGTTGCCCTGGTTCGAA
GFPfwd	ATGGCTAGCAAAGGAGAAGA
GFPrev	TTATTTGTAGAGCTCATCCA
Rna4wtrev	AGGCACACTGAGACGCAAAA
Rna42rev	GAATTCTCTCAGAATCACTA
Rna46rev	AAGCTTTCTCAGAATCACTA
Rna48rev	AAGCTTCTGCAGAATTCGCC
CPfwd	ATGGACAAATCTGGATCTCCCAAT
CPrev	CTAAGTCGGGAGCATCCGTGAGAT
EATG	CTGAGAGAATTCATGGACAAATCTGGATCTCCCAAT
HATG	CTGAGAAAGCTTATGGACAAATCTGGATCTCCCAAT
PHATG	GAGACTGCAGAAGCTTATGGACAAATCTGGATCTCCC
RNA1fwd	GTTTTATTTACAAGAGCGTA
RNA12rev	TGGTCTCCTTTTGGAGGCC
RNA2fwd	GTTTATTCTCAAGAGCGTA
IRNA12fwd	GTTTATTTACAAGAGCGTACGGTT
IRNA12rev	TGGTCTCTTTTAGAGGCCCCCACG
downstreamfwd	TCCGTGTGTTTACCGGCGTCCGAA
endoonlyfwd	ATGAAGAATACATCCTCCTT
endoonlyrev	TCAGTGGTGATGGTGATGATGGGA
35SPfwd	GTCAACATGGTGGAGCACGA
35STrev	GATTTTAGTACTGGAT
35SPrev	CCTCTCCAAATGAAATGAAC
35STfwd	ATTCGGTACGCTGAAATCAC
33delfwd	TAGTGTTTTGTTACGTTGTACCT
33delrev	CCCAGACGCATTTTGATTAAGAG

**Figure 1:**
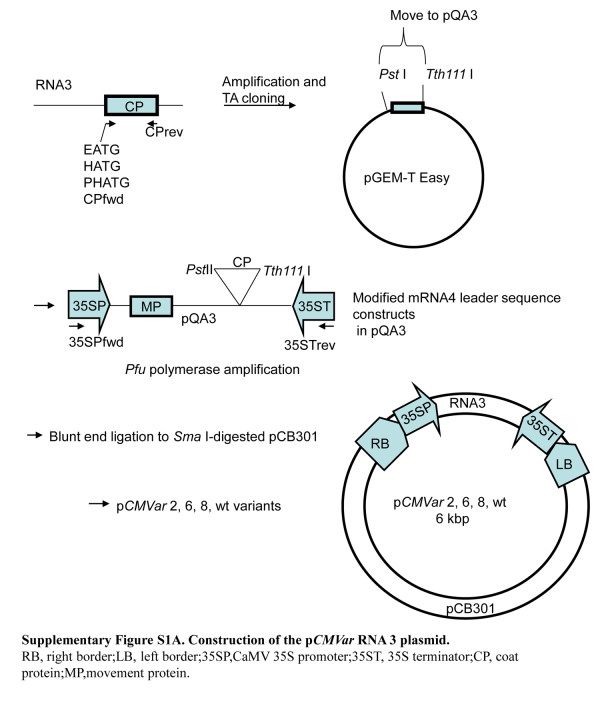
**(A) Diagram of the E1 constructs and (B) Modified RNA 4 leader sequences containing restriction enzyme sites compared to unmodified wild type (wt) leader sequences.** (**A**) Gene structure of the E1 used in this report. The E1 gene contains the rice amylase signal peptide in the upstream of its ORF and a 6 histidine-tag in its C-terminus. RAmySP; rice amylase signal sequence. This Figure is not to scale. (**B**) Restriction endonuclease sequences are shown as *italicized* and underlined, the CP start codon (AUG) is shown in **bold**. Modified leader sequences are located between the wildtype leader sequence and AUG start codon in subgenomic RNA4. Wt, wildtype leader sequence; 2+, modified #2 leader sequences addition to the wildtype leader sequence; 6+, modified #6 leader sequences addition to the wildtype leader sequence; 8+, modified #8 leader sequences addition to the wildtype leader sequence.

The E1 sequence (GenBank:HQ541433) used here was first codon-optimized for dicots and constructed to contain the rice alpha amylase (RAmy 3D, GeneBank:M59351) signal peptide at its N’-terminus, and a 6-His tag at its C’ terminus (synthesized by DNA2.0, Menlo Park, CA, http://www.dna20.com, and provided as plasmid DNA pJL201:11772) ([16], see Figure 1A). The green fluorescent protein (GFP) and E1 coding sequences were PCR amplified and cloned into the CP coding region of p*CMVar* RNA 3.

First, primer sets downstreamfwd and the Rna4wtrev, Rna42rev, Rna46rev, Rna48rev (listed in Table 1), were used for reverse PCR to remove the CP coding region (Additional file 3: Figure S3). The E1 gene was amplified by PCR using specific primers set (endoonlyfwd and endoonlyrev as listed in Table 1) and ligated into coat protein gene-deleted p*CMVar* RNA 3 by blunt end ligation (Additional file 3: Figure S3), yielding p*CMVar* E. The GFP coding sequence was PCR amplified from p*CMViva* GFP [9] using the specific primer sets (GFPfwd and GFPrev listed in Table 1), and cloned into the coat protein region of p*CMVar* RNA 3 using the same methods as for E1, resulting in p*CMVar* G (Additional file 3: Figure S3).

To generate the *CMVar* replicating constructs, CMV RNA 1 and RNA 2 segments (for CMV subgroup I and II) were PCR amplified using the specific forward and reverse primer sets (RNA1fwd and RNA12rev for subgroup II RNA 1, RNA2fwd and RNA12rev for subgroup II RNA 2, IRNA1fwd and IRNA12rev for subgroup I RNA 1, IRNA2fwd and IRNA12rev for subgroup II RNA 2, respectively, as listed in Table 1). The RNA 3 region of p*CMVar* RNA 3 was removed and replaced by the RNA 1 or 2 genome segments and gave I and II p*CMVar* RNA 1 and 2 (Additional file 4: Figure S4). The subgroup I RNA 1 and RNA 2 were originally from a California CMV [17], and the subgroup II RNA 1 and RNA 2 were from CMV-Q (GenBank:X02733 for RNA 1, X00985 for RNA 2, respectively).

For the *CMVin* system, RNA 1 and 2 segments came from pDUXLR1R2 (pR1R2; [9] which includes the modified RNA 1 sequence. The monopartite inducible *CMViva* expression system, pCMV containing all three CMV genomic RNA segments in a single plasmid was used as control [9]. The plasmid, pCassQ123, containing all three CMV RNA segments in a single plasmid and each driven by the 35S promoter was a gift from Dr. ShouWei Ding, UC Riverside.

In order to construct the CMV MP 33 amino acid deletion mutants, we used PCR and the specific primer set (33delfwd and 33delrev, listed in Table 1). PCR products were eluted from an agarose gel and self ligated to make p*CMVar* 33 G 2, 6, 8, wt and p*CMVar* 33E 2, 6, 8, wt variants, respectively (Additional file 5: Figure S5). Table 2 shows the names, activities and genotypes for the expression system variants used in this paper.

**Table 2:** Nomenclature and composition of CMV-based systems

**Leader sequences in mRNA4**	**Protein encoded by RNA3**^**1**^	**Genotype of RNA 1 and 2**^**2**^	**Replicating (R) or inducible(I)**	**Name of combined CMV variants**
wt	CP	Subgroup I	R	*CMVar*I wt
2				*CMVar*I 2
6				*CMVar*I 6
8				*CMVar*I 8
wt	CP	Subgroup II	R	*CMVar*II wt
2				*CMVar*II 2
6				*CMVar*II 6
8				*CMVar*II 8
wt	CP	Subgroup II	I	*CMVin*II wt
2				*CMVin*II 2
6				*CMVin*II 6
8				*CMVin*II 8
wt	GFP	Subgroup I	R	*CMVar*I Gwt
2				*CMVar*I G2
6				*CMVar*I G6
8				*CMVar*I G8
wt	GFP	Subgroup II	R	*CMVar*II Gwt
2				*CMVar*II G2
6				*CMVar*II G6
8				*CMVar*II G8
wt	GFP	Subgroup II	I	*CMVin*II Gwt
2				*CMVin*II G2
6				*CMVin*II G6
8				*CMVin*II G8
wt	E1	Subgroup I	R	*CMVar*I Ewt
2				*CMVar*I E2
6				*CMVar*I E6
8				*CMVar*I E8
wt	E1	Subgroup II	R	*CMVar*II Ewt
2				*CMVar*II E2
6				*CMVar*II E6
8				*CMVar*II E8
wt	E1	Subgroup II	I	*CMVin*II Ewt
2				*CMVin*II E2
6				*CMVin*II E6
8				*CMVin*II E8
wt	GFP	Subgroup I	R	*CMVar*I 33Gwt
2				*CMVar*I 33 G2
6				*CMVar*I 33 G6
8				*CMVar*I 33 G8
wt	GFP	Subgroup II	R	*CMVar*II 33Gwt
2				*CMVar*II 33 G2
6				*CMVar*II 33 G6
8				*CMVar*II 33 G8
wt	GFP	Subgroup II	I	*CMVin*II 33Gwt
2				*CMVin*II 33 G2
6				*CMVin*II 33 G6
8				*CMVin*II 33 G8
wt	E1	Subgroup I	R	*CMVar*I 33Ewt
2				*CMVar*I 33E2
6				*CMVar*I 33E6
8				*CMVar*I 33E8
wt	E1	Subgroup II	R	*CMVar*II 33Ewt
2				*CMVar*II 33E2
6				*CMVar*II 33E6
8				*CMVar*II 33E8
wt	E1	Subgroup II	I	*CMVin*II 33Ewt
2				*CMVin*II 33E2
6				*CMVin*II 33E6
8				*CMVin*II 33E8

^1^All RNA3 constructs are from subgroup II CMV-Q. ^2^RNA 1 and 2 sources are subgroup I or subgroup II as indicated. Cells containing required plasmids were mixed and infiltrated into non-transgenic *N. benthamiana* plants as described.

### Agroinfiltration

Binary plasmids purified from *E. coli* cultures were transformed into *Agrobacterium tumefaciens* GV3101 or EHA105 cells using electroporation. Transformed *A. tumefaciens* cells were plated on Luria-Bertani plates containing Rifampicin (10 μg/ml) and Gentamycin (20 μg/ml) for GV3101 and Kanamycin (50 μg/ml) for specific constructs, and Rifampicin (10 μg/ml) and Tetracycline (10 μg/ml) for EHA105 and Gentamycin (20 μg/ml) for specific constructs, respectively. For agroinfiltration, a single colony was inoculated into 5 ml L-MESA media (100 ml LB broth, 2 ml 0.5 M MES (pH 5.7), 20 μl 0.1 M acetosyringone) and grown to an OD_600_ of 1.0. Cells were harvested by centrifuging for 10 min at 3,500 *g* and resuspended in induction media (50 ml sterile dH_2_0, 0.5 ml 1 M MgCl_2,_ 1 ml 1 M MES (pH 5.7), 50 μl 0.1 M Acetosyringone), and allowed to sit at room temperature for 3 hrs before infiltration. When mixtures of *A. tumefaciens* cells were infiltrated into plants, cultures were prepared separately in induction medium and combined immediately before infiltration. For inoculating small sugar pumpkin plants, *A. tumefaciens* cells containing the constructs were infiltrated into *N. benthamiana* plants. Leaves were harvested 6 days after infiltration, and used for standard rub inoculation.

### RNA extraction and realtime RT-PCR

Samples for RNA and protein extraction were harvested from infiltrated and non-infiltrated leaves at 6 days after infiltration. Total RNA was extracted using the RNeasy kit (QIAGEN Inc., U.S.A.) following the manufacturer’s instructions. Complementary DNA (cDNA) synthesized from DNase-digested total RNA was used for reverse transcription using the RNA 3end primer as listed in Table 1 and SuperScript II Reverse Transcriptase, as described by the manufacturer (Invitrogen, Carlsbad, CA, U.S.A.). Realtime PCR was performed using gene specific primers for each CMV RNA segment (realrna1fwd and realrna1rev for RNA 1, realrna2fwd and realrna2rev for RNA 2, realrna3onlyfwd and realrna3onlyrev for RNA 3, realrna4onlyfwd and realrna4onlyrev for RNA 4, and real18Sfwd, real18Srev for endogenous 18S control, respectively as listed in Table 1). Real-time PCR was performed using SYBR Green PCR master mix (Applied Biosystems, Life Technologies Corporation, Carlsbad, CA, U.S.A.) in an ABI Prism 7500 Sequence Detection system (Applied Biosystems, Life Technologies Corporation, Carlsbad, CA, U.S.A.) under standard amplification conditions (95°C for 5 min, followed by 40 cycles of 95°C for 15 s and 60°C for 1 min). The threshold cycle (*C*_T_) is defined as the fractional cycle number at which the fluorescence exceeded the fixed threshold. Statistical analyses were performed using the Bonferroni (Dunn) t test using the SAS 9.1 program.

### Protein extraction and immunoblotting

Proteins were extracted from leaves using protein extraction buffer (100 mM Tris–HCl (pH 8.0), 10 mM EDTA, 5 mM DTT, 150 mM NaCl, 0.1% Triton X-100, 1X protease inhibitor (Roche diagnostics, Germany) and tissue maceration using a bead-beater. Samples were centrifuged at 12,000 *g* for 20 min to remove cell debris, and protein concentrations were determined by Bradford assay using Coomassie Plus (Pierce, Thermo Scientific, IL, U.S.A.) with bovine serum albumin as the standard. Proteins were analyzed by SDS-PAGE in 12% polyacrylamide gels and transferred to Hybond-C Extra membranes (Amersham Pharmacia Biotech, U.K.). Membranes were incubated with rabbit CMV anti-CP polyclonal antibody at 1:2,500 dilution, followed by goat anti-rabbit IgG-alkaline phosphatase conjugate (Bio-Rad, Hercules, CA, U.S.A.) at 1:2,500 dilution. For E1 detection, membranes were incubated with specific mouse monoclonal IgG anti-E1 antibody (provided by Bill Adney, National Renewable Energy Lab) at 1:2,500 dilution, followed by goat anti-mouse IgG alkaline phosphatase conjugate (Bio-Rad, Hercules, CA, U.S.A.) at 1:2,500 dilution. After washing with Tris-buffered saline (100 mM Tris-Cl, pH 7.5, 0.9% NaCl) with 0.3% Tween-20 for three times, the membrane was developed to a purple color using colorimetric AP conjugate substrate reagent kit (Bio-Rad, Hercules, CA, U.S.A.) including premixed BCIP (5-bromo-4-chloro-3-indolyl phosphate) and NBT (nitroblue tetrazolium) substrate solutions (Bio-Rad, Hercules, CA, U.S.A.).

### Endoglucanase (E1) activity assays

Endoglucanase (E1) activity assays were done as described [16]. A 60 μl aliquot of diluted supernatant containing E1 was added to 540 μl of acetate buffer (200 mM acetate, pH 5.5, 100 mM NaCl) and 200 μl of substrate (500 μM methylumbelliferyl-tagged cellobiose (MUC)). Then, 200 μl of the reaction was sampled at time zero and after 30 min, and added to 800 μl of stop buffer (150 mM glycine, pH 10). The change in fluorescence as released methylumbelliferine (MU) over time was measured with a VersaFluor fluorometer (Bio-Rad, Hercules, CA, U.S.A.). Fluorescence was converted to activity, and specific activity was determined as described [16].

## Results

### Modified RNA4 leader sequences affect mRNA and coat protein levels

To facilitate cloning desired genes into the CMV CP region, we first created a separate CMV RNA 3-based plasmid and modified the intergenic nucleotide sequence upstream of the CP ORF start codon to contain desired restriction endonuclease sites (Figure 1). This also resulted in changes to the mRNA 4 nucleotide leader sequence immediately preceding the AUG start codon and therefore could affect mRNA translation efficiency [18-20]. Therefore, we first compared the respective *CMVin* and *CMVar* variants for their ability to replicate and to express CMV CP within infiltrated leaves. We used real-time PCR to quantify levels of progeny RNAs 1, 2, 3, and 3 plus 4 (because RNA 4 is a subset of RNA 3 and is therefore difficult to differentiate from RNA 3).

**Figure 2:**
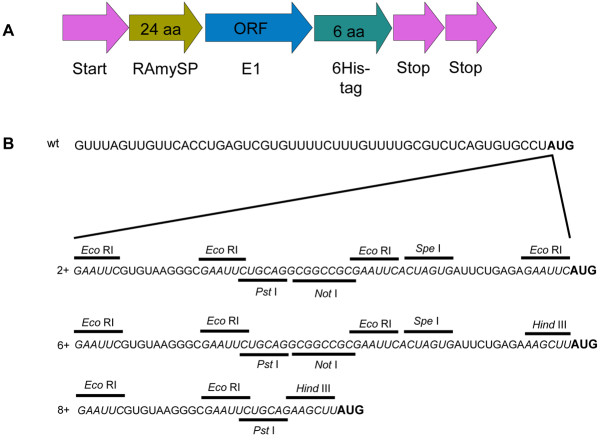
**Relative RNA and coat protein accumulation levels for *CMVin*II and *CMVar*II variants having wt or modified RNA 4 leader sequences.** After 6 days post-infiltration, infiltrated leaves were harvested and total RNAs and proteins were extracted as described in Methods. For *CMVin*II variants (see Table 2), estradiol was applied 18 hrs after infiltration using a cotton swab. For *CMVin*II variants, two leaves each from 3 plants for each treatment (n = 6) were used for 1^st^, 2^nd^ biological trials, and three leaves each from 2 plants for 3^rd^, 4^th^ trials (n = 6) yielding four different biological trials (total n = 24). For *CMVar*II variants (see Table 2), two leaves each from 3 plants for each treatment (n = 6) were used for analysis. These experiments were replicated three times (total n = 18). For immunoblot analysis, each lane was loaded with 15 μg of total protein. Real-time PCR reactions were performed using an ABI 7500 with gene specific primer sets. Relative Ct values were calculated to the respective 18S rRNA relative concentration. Relative folds were calculated to the repective wildtype *CMVin*II and *CMVar*II value, respectively. Group superscript letters next to the numbers represent different statistical groups, means with the same letter are not significantly different by the Bonferroni (Dunn) t test using the SAS 9.1 program. Panel (**A**) *CMVin*II variants; H , healthy uninfiltrated leaf; pR1R2, p*CMVin*II RNA 1 and RNA 2 only; *CMVin*II wt , wt leader; *CMVin*II 2 , #2 leader; *CMVin*II 6, #6 leader; *CMVin*II 8 , #8 leader; *CMViva*, pCMV (see Methods). Panel (**B**) *CMVar*II variants; H, healthy, uninfiltrated leaf; R1 + R2, p*CMVar*II RNA 1 and RNA 2 only; *CMVar*II wt , wild type leader; *CMVar*II 2 , #2 leader; *CMVar*II 6 , #6 leader; *CMVar*II 8, #8 leader; pQ123, pCassQ123 (see Methods).

When we compared the *CMVin*II variants with *CMViva*, all showed accumulation of genomic and subgenomic RNAs, and of CMV CP (Figure 2A). Because we are most interested in protein production from RNA 4, comparison of the RNA 3 & 4 data show that the modified variants (*CMVin*II 2, 6 and 8) all showed slightly more RNAs 3 and 4 than did the wildtype *CMVin*II, but none were higher than *CMViva* (Figure 2A). Although the levels of RNAs 3 and 4 were not statistically different, comparison of CMV CP accumulation showed more CMV CP was detected for *CMVin*II variants 2, 6, 8 and *CMViva*, than was for the wildtype *CMVin*II. *CMViva* and *CMVin*II variants both have 46 nucleotide deletions in the inducible CMV RNA 1 such that although it is transcribed and the resulting RNA 1 serves for translation to yield the 1a protein, the RNA 1 genome segment is not replicated as are genome segments RNAs 2 and 3 (Additional file 2: Figure S2). Because the 1a component of the replicase complex is under the control of a relatively tightly regulated chemically inducible promoter, the recombinant viral amplicons are only produced under induction conditions.

When we compared the *CMVar*II variants, all showed accumulation of all CMV RNAs, and so long as inocula contained all three CMV genomic RNAs, accumulation of RNAs 1, 2 and 3 was not significantly different for the different variants. However, realtime PCR analysis showed lower accumulation of RNAs 3 + 4 for *CMVar*II variant6 when compared to the monopartite pQ123 (Figure 2B). All *CMVar*II variants showed much less CP accumulation than was seen for pQ123, and *CMVar*II 2, 6 and 8 showed less CP than did *CMVar*II *wt* (Figure 2B). However, all *CMVar*II variants were able to initiate systemic infections in *N. benthamiana* and zucchini squash plants (Additional file 6: Figure S6). RT-PCR and nucleotide sequence analysis of the CMV progeny showed that the modified leader sequences were retained in RNAs extracted from systemically-infected leaves (data not shown).

### *CMVin*II and *CMVar*II variants yield high GFP fluorescence

**Figure 3:**
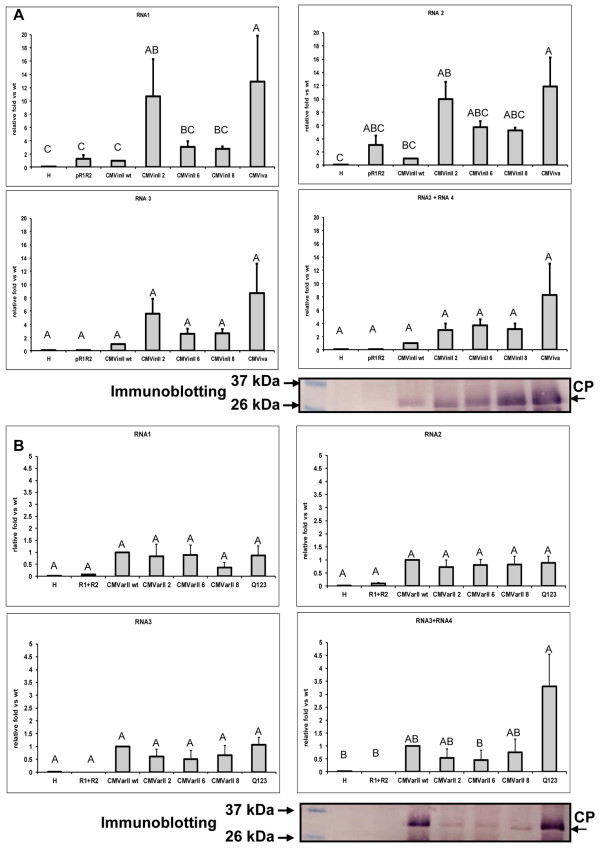
**GFP fluorescence for *CMVin*II and *CMVar*II wt and leader sequence variants in *N. benthamiana *plants.***CMVin*II G and *CMVar*II G (see Table 2) columns are indicated for both 6 and 10 days after infiltration. Rows show relative GFP fluorescence for leader sequence variants. *A. tumefaciens* cells containing respective plasmids were mixed just before leaf infiltration. *CMVin*II G plants then had estradiol induction treatments 18 hrs after infiltration. Photos were taken under long wavelength UV light.

To assess foreign protein production we first cloned the gene for GFP into the CP coding region for the *CMVin*II and *CMVar*II variants. Non-transgenic *N. benthamiana* plants were infiltrated and leaves were examined for fluorescence at 6 and 10 days post-infiltration. At 6 days post-infiltration bright GFP fluorescence was seen in the infiltrated regions (Figure 3). In general, the regions of the leaves infiltrated with *CMVin*II and *CMVar*II variants showed very bright fluorescence. By 10 days post-infiltration, bright GFP fluorescence was observed for the *CMVin*II and *CMVar*II variants, regardless of leader sequence. Despite the fact that *CMVar*II variants producing the CMV CP spread within plants giving systemic infections, *CMVar*II variants producing GFP did not, and fluorescence was localized to the infiltrated areas. This is most likely because these variants do not produce CMV CP, which is known to be a determinant of CMV systemic spread in plants [21,22], and thus these infections were localized to the infiltrated regions of the treated leaves. We also tested another reporter protein, the red fluorescent protein (RFP), for expression using *CMVin*II and *CMVar*II and obtained essentially identical results to those shown for the variants expressing GFP (data not shown).

### E1 was produced in plants using both *CMVin*II and *CMVar*II variants

**Figure 4:**
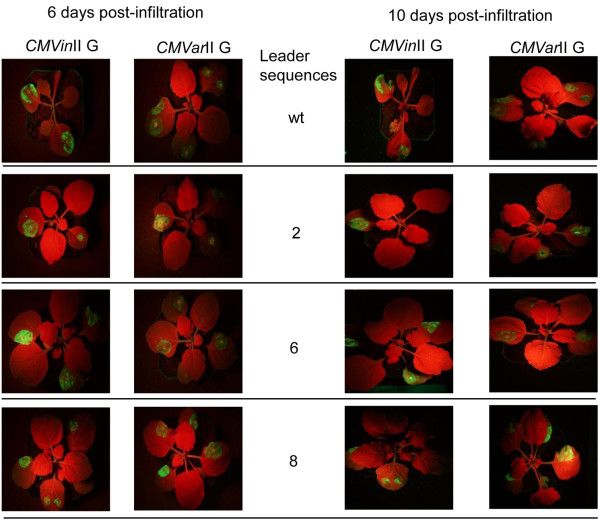
**Immunoblot analysis of E1 produced in infiltrated *N. benthamiana *leaves. ***N. benthamiana* leaves were co-infiltrated with *A. tumefaciens* containing plasmids corresponding to the *CMVin*II E and *CMVar*II E variants (see Table 2) shown. *CMVin*II E leaves were treated with estradiol 18 hrs after infiltration. Leaves were harvested 6 days post-infiltration. Soluble proteins were extracted as described in Methods and concentrations were determined by Bradford assay. Numbers above each lane correspond to the specific RNA 3 variant used. Each sample represents 15 μg total protein extract per lane. Prestained size marker (Benchmark, Invitrogen, Carlsbad, CA, U.S.A.) shows 80 kDa band.

Our intent is to develop easy-to-use CMV variants that give efficient production of desirable proteins in non-transgenic plants, including proteins with potential biofuel applications. Therefore, we next assessed E1 accumulation in leaves of infiltrated *N. benthamiana* plants. We first used immunoblot analysis to detect total E1 accumulation in leaves infiltrated with the *CMVin*II and *CMVar*II variants. E1 was detected in the infiltrated leaves for both *CMVin*II and *CMVar*II wild type variants (Figure 4). The leader sequence *CMVin*II variants 6 and 8 showed higher E1 compared to the wildtype *CMVin*II. For *CMVar*II variants, the wild type showed higher E1 accumulation, but the 6 and 8 variants also gave good E1 accumulation (Figure 4). Interestingly, the intact E1 migrated as a ca. 72 KDa protein as shown in Figure 4 and Additional file 7: Figure S7, even though the calculated MW of E1 is 57.3 KDa, including the histidine tag and rice amylase signal peptide (See Figure 1A). Similar reports for anomalous E1 migration in SDS-PAGE have been previously reported [16,23].

The immunoblot experiments showed E1 protein accumulation but our interest is in production of enzymatically active E1. Therefore we used activity assays on plant extracts to estimate yields of active E1. In repeated experiments, the *CMVin*II E6 and E8 consistently yielded 8 to almost 15 fold higher relative E1 accumulation than wildtype *CMVin*II E, while the yield for *CMVin*II E2 was negligible. By contrast, the wildtype *CMVar*II E consistently yielded more E1 than the other *CMVar*II variants (Table 3). *CMVar*II E2 gave negligible E1 accumulation while *CMVar*II E6 and E8 showed low yields, but much less than wildtype *CMVar*II E. We had anticipated that *CMVar*II would give higher protein accumulation in plants because of RNA 1 replication, which is lacking in *CMVin*II (Additional file 2: Figure S2), but this proved not to be the case, the highest overall yields were obtained with *CMVin*II E 6 and 8. The *CMVar*II is easier to use since there is no requirement for adding the RNA 1 inducer, estradiol, and if we could achieve higher protein accumulation with *CMVar*II this would be our choice. Therefore we next attempted to increase *CMVar*-driven protein accumulation by two additional approaches: to increase CMV RNA replication and to increase CMV spread within plants.

**Table 3:** **Amount of active E1 (μg/g FW) in infiltrated *N. benthamiana *leaves using the different *CMVin *and *CMVar *leader sequence variants**

	**-wt**	**−2**	**−6**	**−8**
*CMVin*II E	1.09 ± 0.20^1D^	−0.01 ± 0.00^D^	14.74 ± 2.14^A^	13.97 ± 3.40^A^
	1.07 ± 0.80^E^	0.22 ± 0.36^E^	8.76 ± 3.49^BC^	9.38 ± 2.10^BC^
	1.00 ± 0.45^D^	0.76 ± 0.11^D^	10.05 ± 1.28^B^	11.05 ± 3.85^B^
*CMVar*II E	2.23 ± 0.59^CD^	0.00 ± 0.01^D^	0.10 ± 0.08^D^	−0.01 ± 0.00^D^
	6.30 ± 3.50^CD^	0.01 ± 0.03^E^	0.33 ± 0.33^E^	0.05 ± 0.12^E^
	10.78 ± 3.63^BC^	−0.01 ± 0.01^D^	1.58 ± 0.49^D^	1.85 ± 1.13^D^
*CMVar*I E	4.12 ± 3.35^CD^	0.00 ± 0.01^D^	0.67 ± 0.36^D^	−0.01 ± 0.00^D^
	9.38 ± 2.10^AB^	0.00 ± 0.01^E^	0.57 ± 0.45^E^	0.38 ± 0.79^E^
	15.15 ± 5.39^AB^	0.39 ± 0.30^D^	1.88 ± 0.32^D^	1.28 ± 0.53^D^
*CMVar*II 33E	6.74 ± 2.82^BC^	0.08 ± 0.12^D^	1.17 ± 0.82^D^	2.27 ± 1.81^D^
	5.61 ± 2.01^CDE^	0.01 ± 0.01^E^	1.87 ± 0.64^DE^	1.15 ± 0.77^DE^
	14.06 ± 2.71^AB^	0.21 ± 0.26^D^	1.17 ± 0.98^CD^	1.72 ± 0.93^CD^
*CMVar*I 33E	11.34 ± 2.67^AB^	1.42 ± 0.33^D^	2.26 ± 0.36^CD^	1.31 ± 0.81^CD^
	19.60 ± 4.56^A^	1.49 ± 0.14^DE^	2.03 ± 0.31^CE^	1.73 ± 1.09^DE^
	20.99 ± 5.18^A^	1.16 ± 0.96^D^	1.97 ± 0.48^D^	2.49 ± 1.61^D^

^1^Numbers indicate E1 production as μg/g fresh weight. Average ± standard deviations are shown. Samples were collected 6 days post-infiltration, using 3 plants, 2 leaves from each plant (n = 6) for each biological trial. Results for three different biological trials are shown for each test. The identities of variants tested are given in Table 2. Group superscript letters next to the numbers represent different statistical groups, means with the same letter are not significantly different by the Bonferroni (Dunn) t test using the SAS 9.1 program.

### Reassortant *CMVar*I variants yield more protein compared to *CMVar*II variants

**Figure 5:**
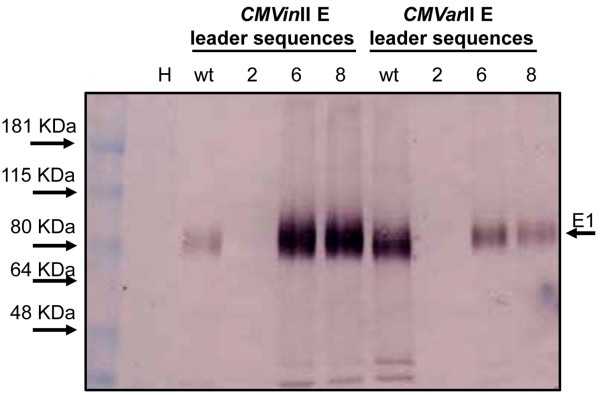
**Comparison of GFP fluorescence from *CMVar*I G and *CMVar*II G variants. ***N. benthamiana* leaves were co-infiltrated with *A. tumefaciens* containing plasmids corresponding to the *CMVar*I and II G variants (see Table 2) as shown at Panel **A**. Numbers for leaves in panel at right indicate specific RNA 3 variant. Photographs were taken 6 days post-infiltration under UV light shown as Panel **B.**

CMV is one of the world’s most widespread plant viruses, and has many genetic variants which are primarily divided into the taxonomic subgroups I and II [5]. In general, subgroup I CMVs show more severe symptoms in plants than do subgroup II CMV isolates, which can show mild or even symptomless infections. This is suggested to be associated with the 2b protein (encoded by RNA 2) as a silencing suppressor [24], and effects can vary in different plant hosts [25]. Therefore, we generated and compared CMV subgroup I and II genomic reassortants for their abilities to give greater replication and protein production. All reassortants contained the same CMV subgroup II wildtype RNA 3 or variant constructs for GFP or E1. GFP fluorescence was brighter for all *CMVar*I variants compared with the respective *CMVar*II variants (Figure 5). We next compared production of active E1 among the *CMVar*I and II E variants by immunoblotting (Additional file 7: Figure S7) and found that the reassortant wildtype *CMVar*I E gave more active E1 than did *CMVar*II E in side-by-side experiments (Table 3 and Additional file 7: Figure S7). By contrast, *CMVar*I E variants 2, 6 and 8 gave very low E1 accumulation. However, wildtype *CMVar*I E gave relatively high E1 accumulation, similar to that for *CMVin*II E variants 6 and 8.

### The MP C-terminal 33 amino acid deletion constructs showed increased yields compared to the intact MP constructs

**Figure 6:**
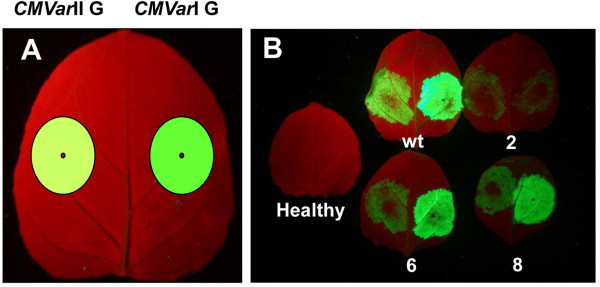
**Comparison of GFP fluorescence *CMVar*I 33 G and *CMVar*II 33 G MP deletion mutants. ***N. benthamiana* leaves were co-infiltrated with *A. tumefaciens* containing plasmids corresponding to the *CMVar*I and II 33 G variants (see Table 2) as shown at left Panel **A**. Numbers for leaves in Panel **B** at right indicate specific RNA 3 variant containing MP deletion. Photographs were taken 6 days post-infiltration under UV light shown as Panel **B.**

CMV requires both the MP and CP for cell-to-cell movement in plants, both of which are encoded by RNA 3 [5,26]. Thus for both *CMVar* and *CMVin* variants*,* when foreign sequences are cloned into the CP coding region, there is no cell-to-cell movement due to lack of the CMV-encoded CP, and the desired recombinant proteins (GFP or E1) accumulate only in the initially-infected cells. However, it was shown previously that when the CMV MP was mutated so as to lack the C-terminal 33 amino acids, CMV infections were able to move cell-to-cell in plants even in the absence of CP [27]. Therefore, we deleted the MP C-terminal 33 amino acids and compared E1 and GFP accumulation in plants using the *CMVar*I and *CMVar*II variants. GFP fluorescence was high for all variants with the 33 amino acid truncated MP (Figure 6). However, comparing E1 accumulation for all variants, the highest levels of active E1 were obtained for the 33 amino acid truncated *CMVar*I Ewt variant (Table 3). Our assays were for intact, enzymatically active E1, and we obtained yields up to 21 μg/g of active E1, corresponding to ~0.4% of TSP. Furthermore, unlike for the wildtype MP variants, the *CMVin*II 33E variants 6 and 8 gave relatively low accumulation of E1.

## Discussion

Several different viruses have been used for protein production in plants, and each has advantages as well as disadvantages [28-32]. Many of the “first generation” plant virus vectors [28,33] utilized whole plant systemic virus infections to give desired proteins. While many of these have proven to be very useful there are some significant drawbacks. Systemic infections can take several days to fully develop. Protein production is then asynchronous and yields can vary in different tissues [3]. Recombinant viruses also show size constraints for the inserted sequence, often coding sequences of only 1 kb or less (encoding a protein of only ~35 kDa) can be inserted [34]. Then as the infection develops the viruses partially or completely excise the inserted recombinant sequence, leading to loss of the desired intact protein product [28,35]. Furthermore, some viruses (e.g. those with icosahedral capsids such as CMV) may have even more severe size constraints if RNA encapsidation is a requirement for development of the systemic infection. Then if the coding sequence for the desired protein is large, insertion into the viral RNA may preclude encapsidation, thereby preventing efficient spread.

Recent progress in developing *Agrobacterium tumefaciens* delivered plant virus-based protein production systems has been made by several research groups using different plant viruses [31,34,36]. These plant virus-based amplicon systems offer many advantages including the fact that non-transgenic plants can be used, the desired protein production is rapid, the product can accumulate to high levels, and virus-based expression can be temporally regulated to be almost synchronous in all infiltrated areas. Because a majority of the infiltrated cells become simultaneously infected, virus movement to new cells is not necessary, encapsidation of recombinant RNAs is not an issue. These “second generation” virus-based systems also can retain larger foreign coding sequences and thus produce larger proteins in plants [34], here we produced enzymatically active 56,000 MW E1.

In our previous work, we used the estradiol-inducible, CMV-based *CMViva* to produce α anti-trypsin (AAT) in non-transgenic *N. benthamiana* plants [9]. *CMViva* has all three CMV genome components in one large 28 kbp plasmid, which, due to its large size is difficult to manipulate. Thus, here we took approaches to develop CMV-based inducible (*CMVin*) as well as autonomously replicating (*CMVar*) systems, both of which are more easily manipulated and might be able to give high accumulation of heterologous proteins in plants. First, we separated the CMV genomic RNA cDNAs onto two plasmids, one containing the RNA 1 and 2 replication-associated genome components and the other containing the CMV RNA 3 genome segment. The CMV RNA3 component is rather small in size, 2.2 kb, and is easy to manipulate and to engineer to contain restriction enzyme sites to allow for easy removal of the CMV CP gene and replace it with any gene of interest. The desired restriction enzyme sites were introduced into the intergenic region of RNA 3. As expected, these altered the 5′ untranslated leader sequence of the resulting mRNA (RNA 4). The sgRNA promoter (for RNA 4 transcription) is within the minus strand of RNA 3 and is recognized by the RNA-dependent RNA polymerase and mRNA transcription is initiated. For CMV-Q RNA3, the transcription initiation starts at nt position 1167 in the intergenic region, which is upstream of the modified leader sequences. Our analyses demonstrated that the RNA 3 modifications affected RNA 3 and RNA 4 accumulation, but showed even more unpredictable effects on resulting protein accumulation. It does not appear that these can be attributed only to start codon context [18,19] as the same construct (RNA 3) showed different protein yields whether the RNA was delivered using *CMVin vs.CMVar*.

In contrast to *CMViva*, both *CMVin*II and *CMVar*I and II variants require mixing *A. tumefaciens* cells containing different plasmids which are then co-infiltrated into plants and T-DNA from the different *A. tumefaciens* cells containing the CMV plasmids must be transferred to the same plant cell for the complete CMV amplicon. For *CMVin*II variants this is then followed by induction using estradiol, which resulted in high level accumulation of the proteins tested here (CP, GFP, E1). However, like for *CMViva,* the *CMVin*II RNA 1 deletion does not allow for its replication, only translation of the newly transcribed mRNA. Therefore we also developed the non-inducible autonomously replicating CMV-based system, *CMVar*I and II. Wildtype *CMVar* (expressing the CMV CP) replicated to very high levels and even caused systemic infections in plants. However, when genes for GFP or E1 were substituted for the CP gene, both proteins were produced in plants within the infiltrated areas, and quantitative analyses showed that high levels of proteins accumulated for both *CMVin vs.CMVar*, particularly at 6 days post-infiltration.

Although *CMVin*II E 6 and 8 variants gave slightly more active E1 in most experiments, *CMVar*I and II variants offers advantages in ease of use (e.g. no need to add the inducer) and thus, two additional approaches to improve accumulation of the desired protein product were investigated. Like most viruses having genomes composed of multiple segments, CMV genomic RNAs can be mixed (reassortment) to achieve genetic diversity [37,38], and this offers opportunities for using CMV to produce desirable proteins in different plant species, as has been demonstrated also by others [3]. Therefore, we generated CMV reassortant genotypes by substituting CMV subgroup I genomic RNAs 1 and 2 derived from a more virulent CMV, with the original CMV Q subgroup II RNA 3, giving *CMVar*I. Comparison of *CMVar*I and *CMVar*II G, E variants showed higher GFP and E1 for *CMVar*I G, E variants. However, the *CMVar*I Ewt showed higher E1 accumulation than did the corresponding *CMVin*II Ewt, but *CMVin*II E variants 6 and 8 gave the higher E1 accumulation than *CMVar* I 6 and 8 variants thus showing that reassortment alone was not sufficient.

As another alternative, we generated a MP C-terminal 33 amino acid deletion mutant. Cell-to-cell movement in CMV-infected plants requires interactions between the CP and MP [22]. Our CMV-based systems including *CMViva*, *CMVar*I and II and *CMVin*II are cell-to-cell movement deficient since they lack the CP and thus, desired recombinant proteins are produced only within infiltrated cells. However, previous workers demonstrated that the CMV MP C-terminal 33 amino acids are essential to recognize and interact with the CP [26]. When this region is deleted, the CMV infections can spread cell-to-cell even in the absence of CP [27]. In support of this the *CMVar*I and *CMVar*II 33 G variants showed high CMV-based GFP production (Figure 6; and see [4]). When we created MP 33 amino acid deletion constructs and tested them, they showed increased production of not only GFP in *CMVar*I and II 33 G variants, but also of E1 in *CMVar*I and II 33E variants (Table 3, Figures 5 and 6), and the highest yields of active E1 were obtained using the *CMVar*I 33E variants.

Other workers have produced versions of E1 in various transgenic plants with gene expression driven by different promoters. For example, full-length E1 containing the catalytic domain, linker and carbohydrate binding domain has been previously produced in transgenic tobacco plants. Based on the resulting E1 activity, yields of up to 0.25% on average of total leaf soluble proteins were shown with Mac promoter, a chimeric promoter of the CaMV 35S and mannopine synthase gene [11]. Similar yields were shown also with CaMV 35S promoter [13]. In transgenic *Z. mays* seeds, the full-length E1 was produced using Glob-1 (Maize embryo-preferred globulin-1 promoter) and yields up to 6% TSP were obtained [39]. In transgenic rice *(Oryza sativa)* plants, 35S driven E1 lacking the carbohydrate binding domain but only containing the catalytic domain gave yields up to 4.9% TSP [40]. Thus, our yields of up to 0.4% TSP of intact E1 in nontransgenic *N. benthamiana* plants are similar to those achieved for intact E1 in transgenic tobacco, but less than those in more specialized systems. Furthermore, *CMVin* and *CMVar*-based production of the desired protein can be temporally regulated to give almost synchronous protein accumulation over a very short time period, even a few days.

## Conclusions

Our data demonstrate that the CMV-based systems, *CMVin* and *CMVar*, are good candidates for production of desired heterologous proteins in nontransgenic plants. Our modifications described here, including manipulating cloning sites for foreign gene introduction, enhance the ease of their use, and reassortant genotypes and CMV movement protein deletions also allow for greater protein accumulation. Also, *N. benthamiana,* which is particularly suitable for agro infiltration, is a very good plant for protein production, but due to the wide host range of CMV, other plants may also prove to be useful for production of different proteins.

## Abbreviations

CMV: *Cucumber mosaic virus*; TMV: *Tobacco mosaic virus; *E1:*Acidothermus cellulolyticus* endo-1, 4-β-glucanase; GFP: Green fluorescent protein; CP: Coat protein; ORF: Open reading frame; MP: Movement protein; FW: Fresh weight; TSP: Total soluble protein; RFP: Red fluorescent protein.

## Competing interests

The authors declare that they have no competing interests.

## Author’ contributions

MSH was responsible for experiment design, execution, analysis and wrote the manuscript. BEL was responsible for experimental execution including enzymatic activity assays and interpretation. KAM helped to conceive the study, discussed, and helped edit the manuscript. BWF helped to conceive the study, helped with organizing the experimental work, data interpretation, and helped to write and edit the manuscript. All authors read and approved the final manuscript.

## Supplementary Material

Additional file 1 **Figure S1.** Construction of the p*CMVar* RNA 3 plasmid. Click here for file

Additional file 2 **Figure S2.** Northern blotting analyses of *CMVin* II variants. Click here for file

Additional file 3 **Figure S3.** Construction of the p*CMVar* G and p*CMVar* E variants for GFP and E1. Click here for file

Additional file 4 **Figure S4.** Construction of the p*CMVar* I and II for subgroup I, II RNA 1 & 2. Click here for file

Additional file 5 **Figure S5.** Construction of the p*CMVar* 33G and p*CMVar* 33E for MP 33 amino acid deletion constructs. Click here for file

Additional file 6 **Figure S6.** Systemic symptoms in plants for *CMVar* II variants. Click here for file

Additional file 7 **Figure S7.** Immunoblot analysis of E1 produced in infiltrated *N. benthamiana* leaves for *CMVar* I E and *CMVar* II E variants. Click here for file
