# Supplementary figures and images for: Bipartite and tripartite Cucumber mosaic virus-based vectors for producing the Acidothermus cellulolyticus endo-1,4-β-glucanase and other proteins in non-transgenic plants
Source: BMC Biotechnol. 2012 Sep 21;12:66. doi: 10.1186/1472-6750-12-66 (PMC3582468; doi:10.1186/1472-6750-12-66)

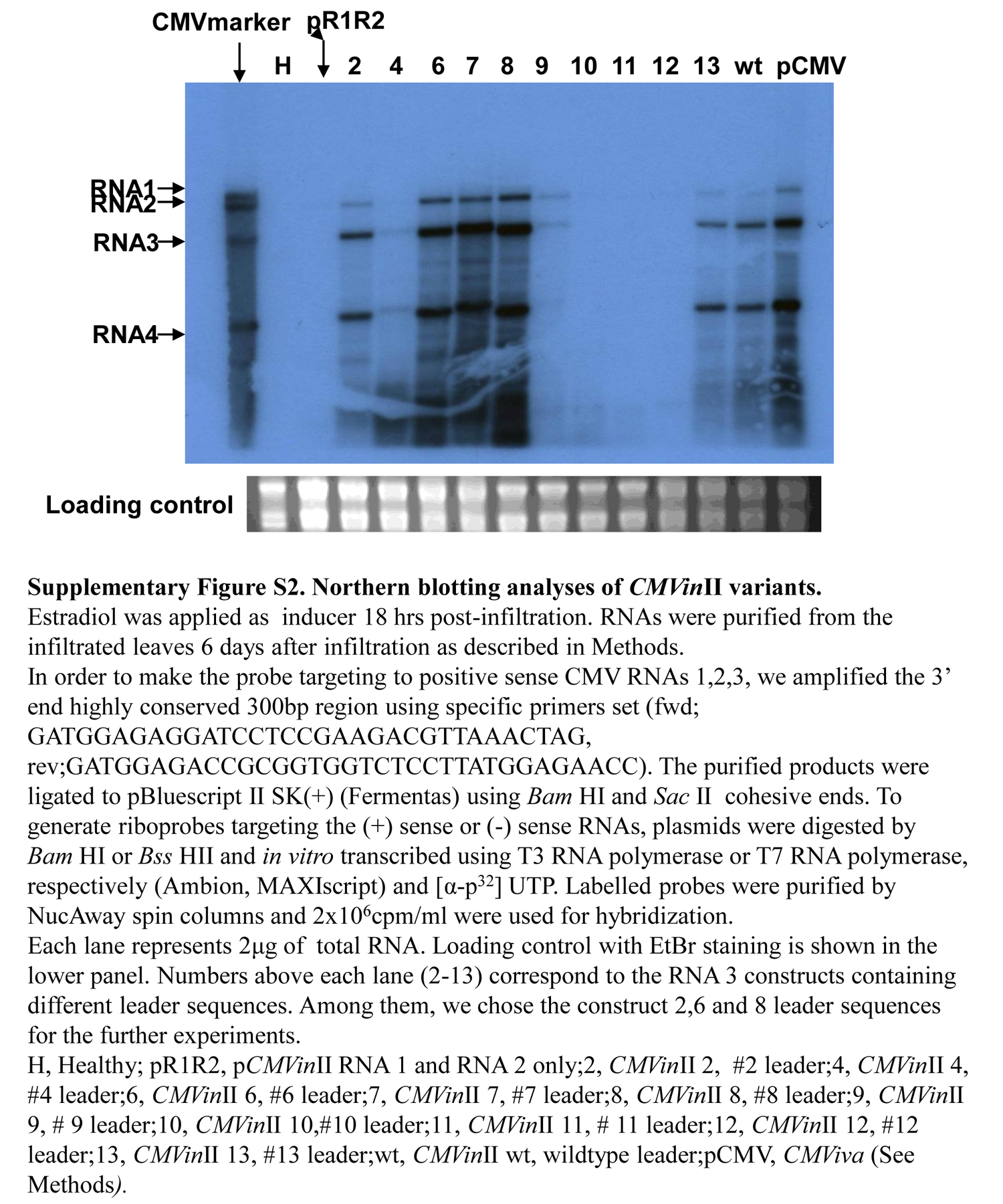

Supplement: Additional file 1 — Figure S1. Construction of the pCMVar RNA 3 plasmid. [file 1472-6750-12-66-S1.tiff]

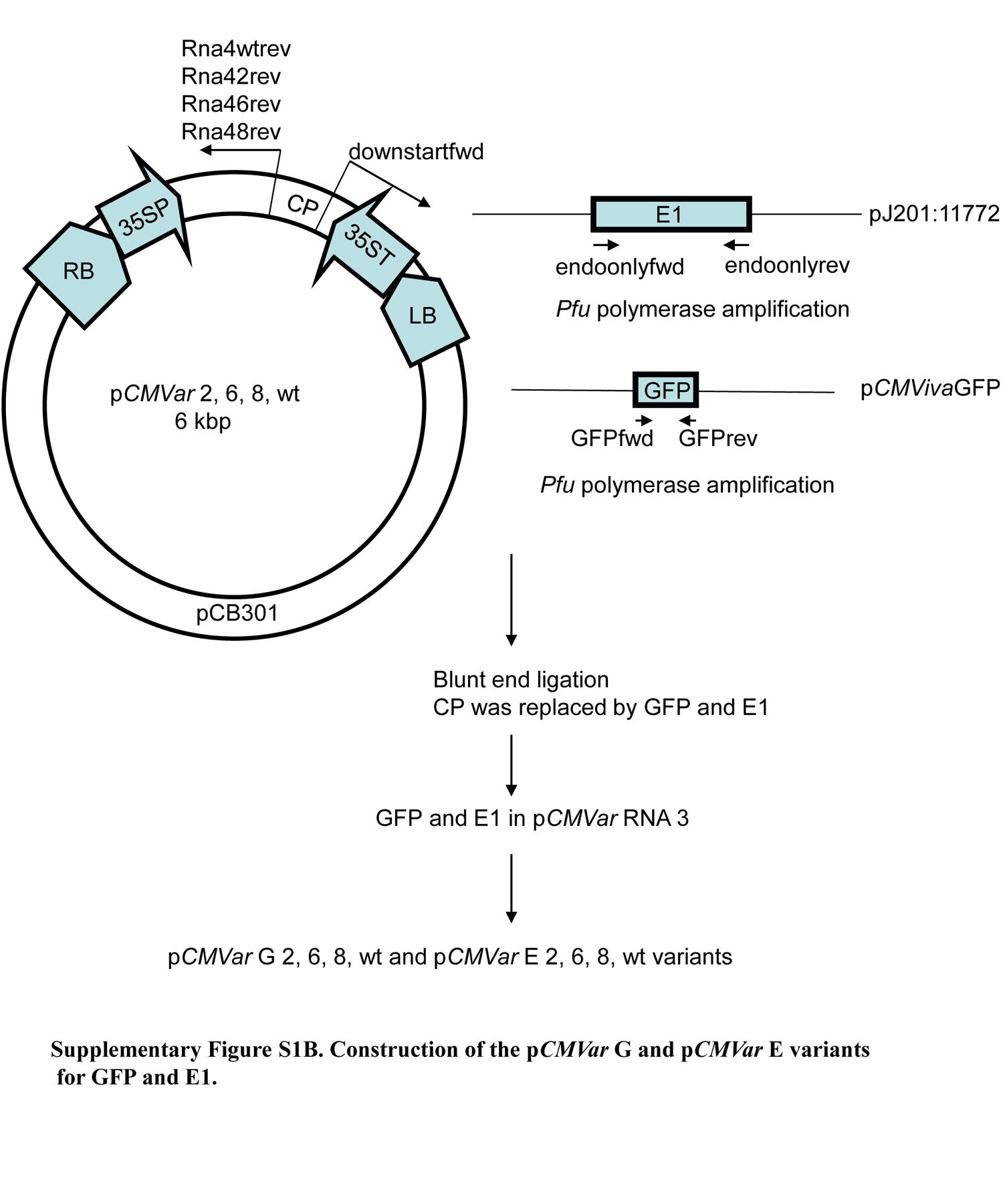

Supplement: Additional file 2 — Figure S2. Northern blotting analyses of CMVin II variants. [file 1472-6750-12-66-S2.tiff]

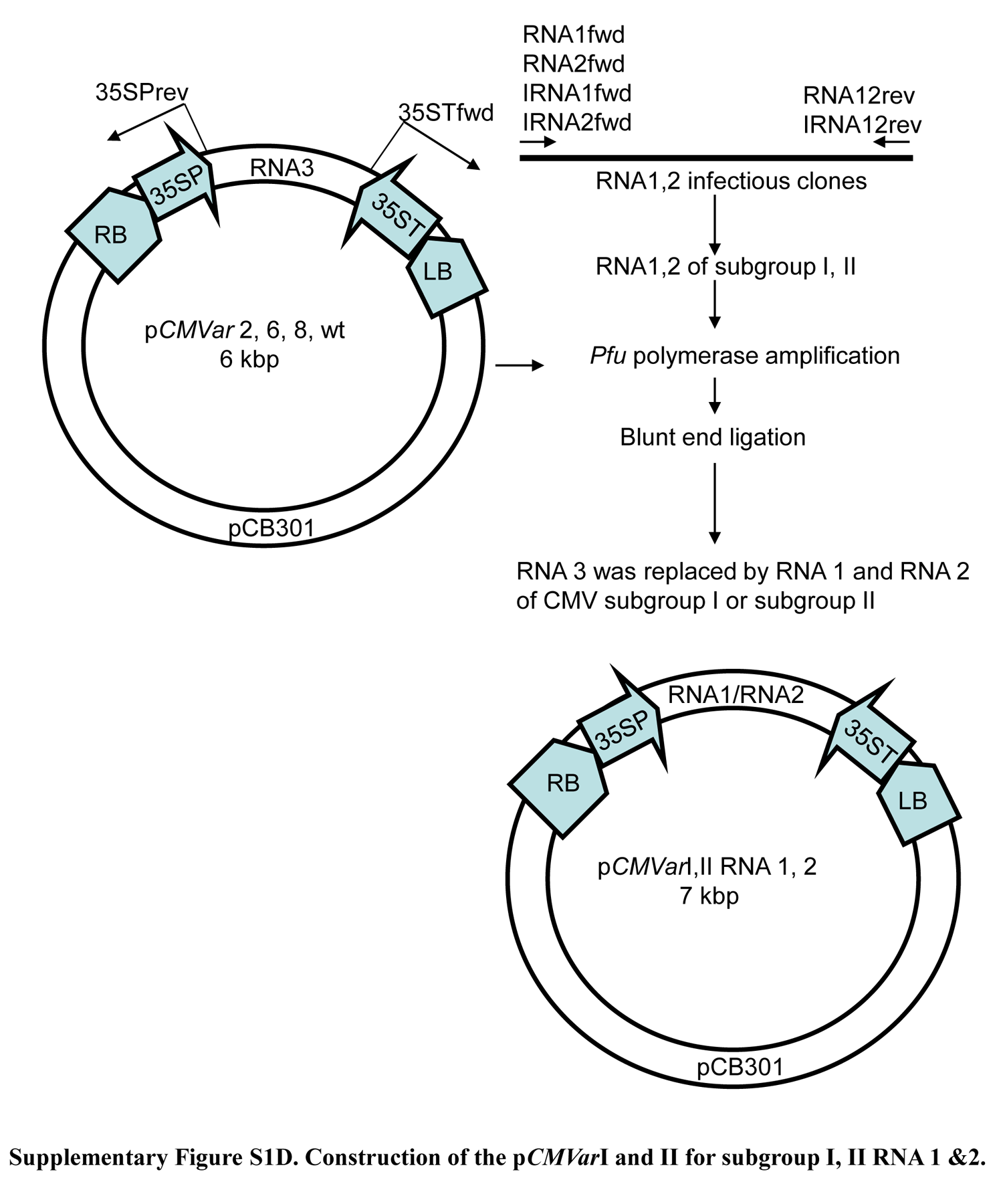

Supplement: Additional file 3 — Figure S3. Construction of the pCMVar G and pCMVar E variants for GFP and E1. [file 1472-6750-12-66-S3.tiff]

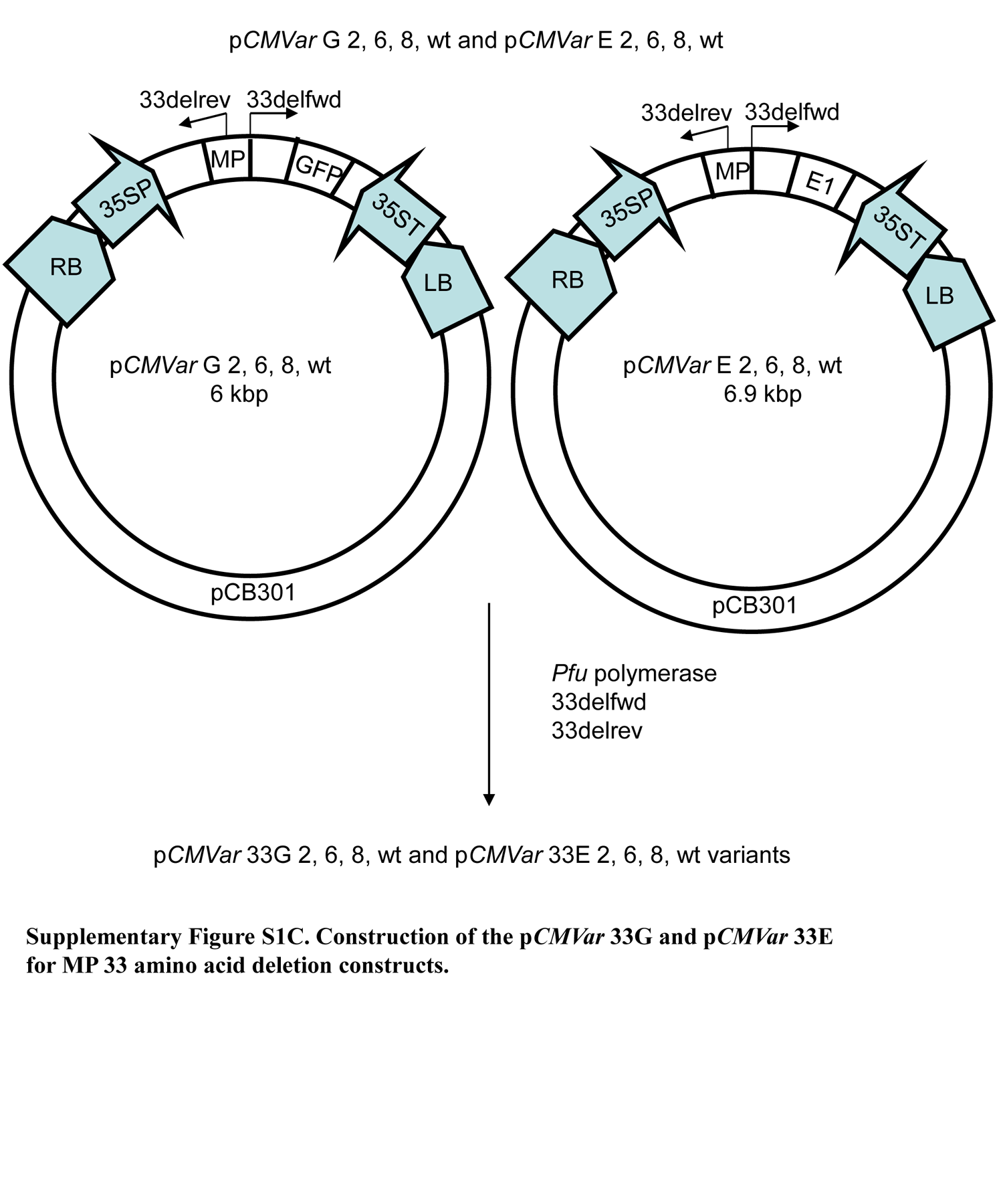

Supplement: Additional file 4 — Figure S4. Construction of the pCMVar I and II for subgroup I, II RNA 1 & 2. [file 1472-6750-12-66-S4.tiff]

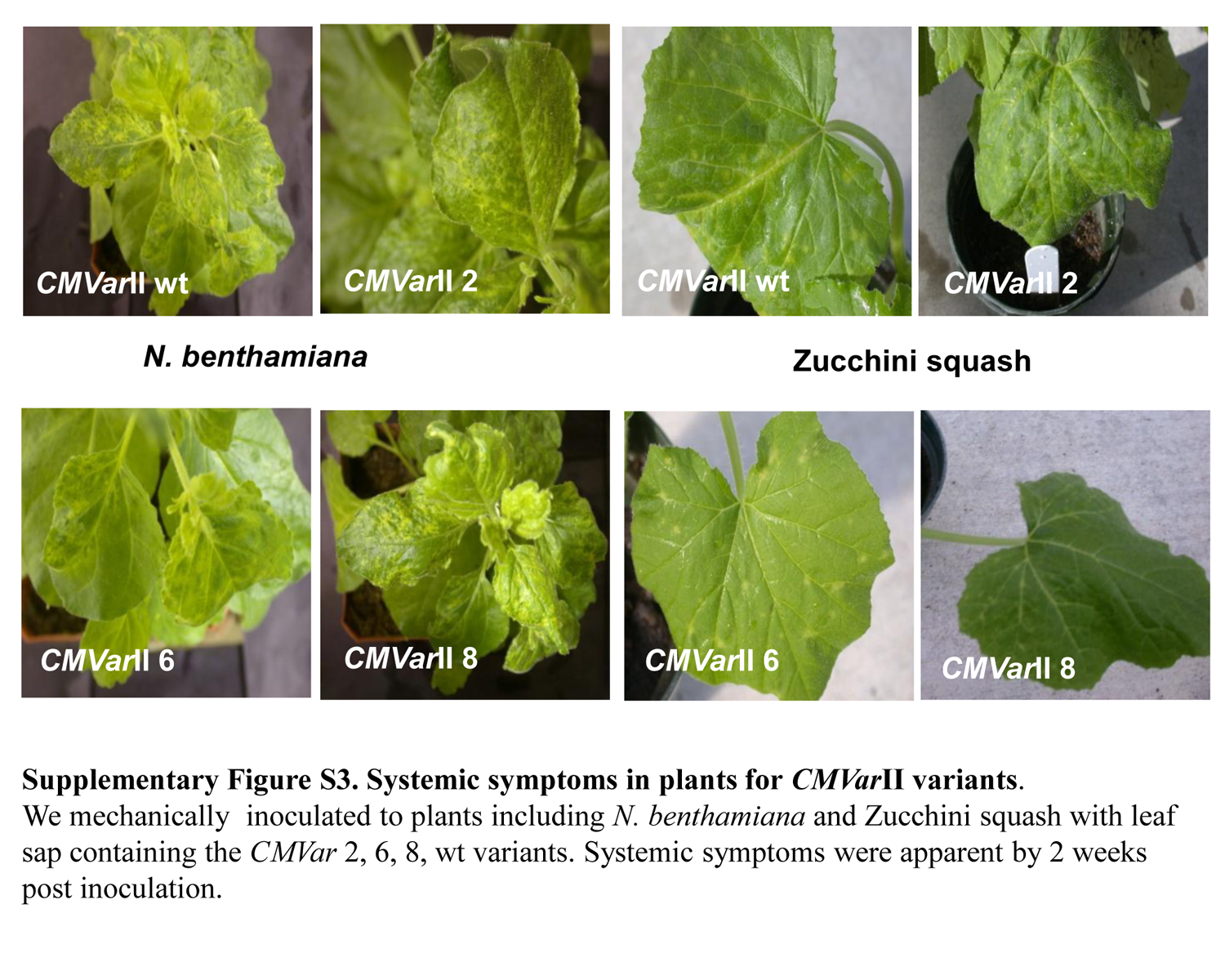

Supplement: Additional file 5 — Figure S5. Construction of the pCMVar 33G and pCMVar 33E for MP 33 amino acid deletion constructs. [file 1472-6750-12-66-S5.tiff]

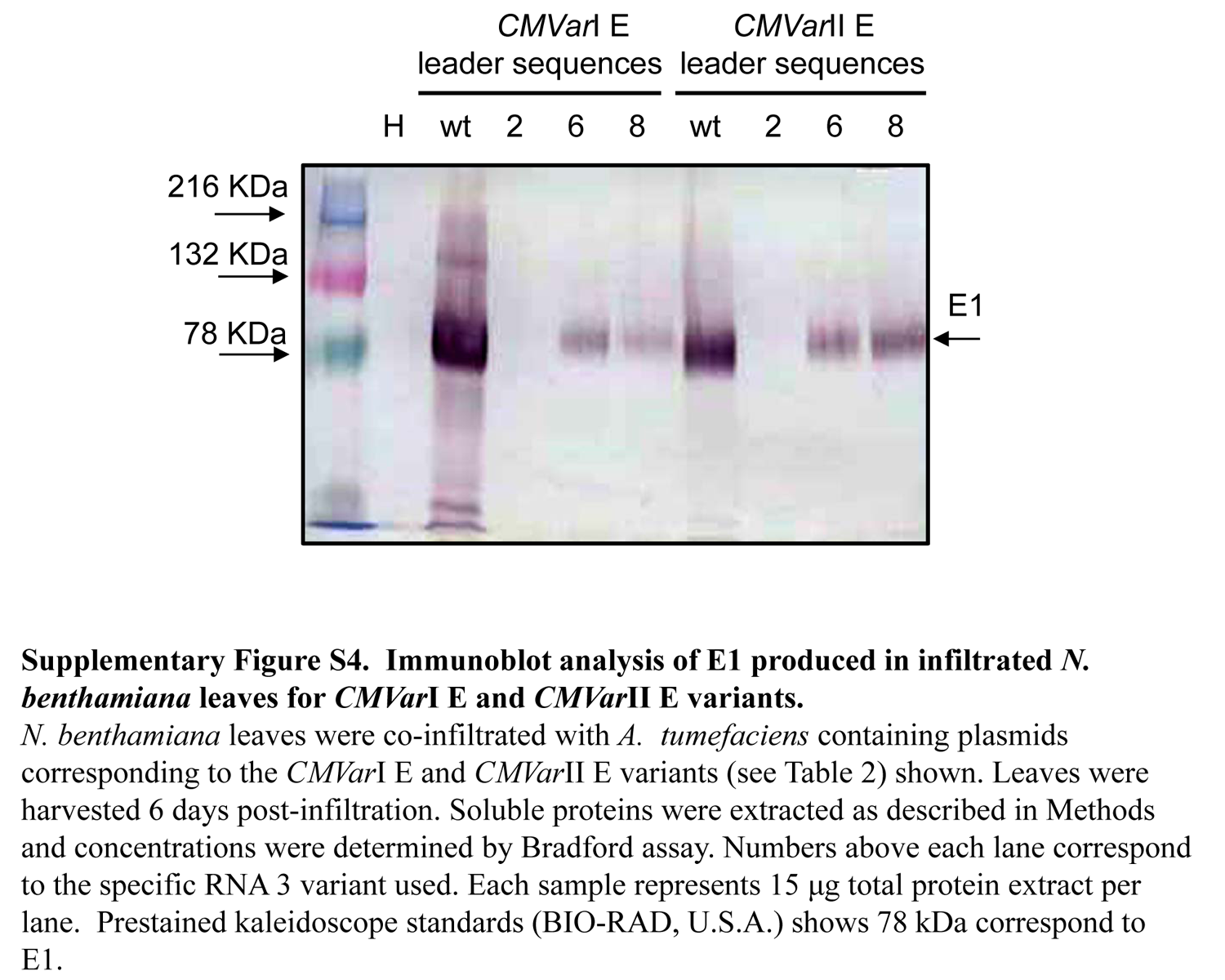

Supplement: Additional file 6 — Figure S6. Systemic symptoms in plants for CMVar II variants. [file 1472-6750-12-66-S6.tiff]
